# The Ethics of Selective Mandatory Vaccination for COVID-19

**DOI:** 10.1093/phe/phab028

**Published:** 2021-12-15

**Authors:** Bridget M Williams

**Affiliations:** Oxford Uehiro Centre for Practical Ethics, Oxford, UK; Center for Population-Level Bioethics, Rutgers University, New Brunswick, USA

## Abstract

With evidence of vaccine hesitancy in several jurisdictions, the option of making COVID-19 vaccination mandatory requires consideration. In this paper I argue that it would be ethical to make the COVID-19 vaccination mandatory for older people who are at highest risk of severe disease, but if this were to occur, and while there is limited knowledge of the disease and vaccines, there are not likely to be sufficient grounds to mandate vaccination for those at lower risk. Mandating vaccination for those at high risk of severe disease is justified on the basis of the harm principle, as there is evidence that this would remove the grave public health threat of COVID-19. The risk–benefit profile of vaccination is also more clearly in the interests of those at highest risk, so mandatory vaccination entails a less severe cost to them. Therefore, a selective mandate would create fairness in the distribution of risks. The level of coercion imposed by a mandate would need to be proportionate, and it is likely that multiple approaches will be needed to increase vaccine uptake. However, a selective mandate for COVID-19 vaccines is likely to be an ethical choice and should be considered by policy-makers.

## Introduction

The emergence of coronavirus disease 2019 (COVID-19) has resulted in substantial harm across the world, both as a direct result of the disease and indirectly as a result of the socio-economic impacts of its change on behaviour and policies used to achieve disease control. Several highly effective COVID-19 vaccines have been developed (Folegatti *et al.*, 2020; National Institutes of Health, 2020; Pfizer, 2020) and roll-out of these vaccines is well underway in many countries (Mathieu, 2021). Rapid resolution of the COVID-19 crisis is important, both for mitigating the direct harms of the disease and for allowing socio-economic recovery to begin. Therefore, a range of policy options to achieve rapid vaccine uptake should be considered. With evidence of vaccine hesitancy in several jurisdictions, voluntary uptake alone may not lead to the vaccine coverage levels required for epidemic control. Indeed, a recent report has estimated the mortality impact of the pandemic to be eight times higher over two years in countries with higher vaccine hesitancy (Mesa *et al.*, 2021). As such, the option of making COVID-19 vaccination mandatory requires consideration.

It has been suggested that a COVID-19 vaccine mandate is likely to be appropriate and legally enforceable in the USA (Reiss and Caplan, 2020), and vaccine mandates have indeed been implemented for certain workers in the USA (The White House, 2021). Savulescu *et al.* (2021) have considered the ethics of mandating vaccines in children; however, the possibility of implementing a selective mandate aimed at those at highest risk of severe COVID-19 has, thus far, received little attention. In this paper I argue that it would be ethical to make the COVID-19 vaccination mandatory for those at highest risk of severe disease, but, while there is limited knowledge of the disease and vaccines, there likely are not sufficient grounds to mandate vaccination for those at lower risk if those at higher risk were protected. I offer several considerations for the potential role of selective mandate in COVID-19 vaccination strategies and defend this proposal from two objections: that any mandate risks worsening vaccine hesitancy and that selective mandate is unjustly discriminatory.

## What Is Mandatory Vaccination and When Should It Be Considered?

Mandates are a form of government coercion; that is, they limit the autonomy of an individual to make a free personal choice by threatening punishment for non-compliance. The level of coercion can vary, from the imposition of bureaucratic hurdles to large fines, community service, restriction on freedom of movement or, in extreme cases, forced vaccination. Although the terms mandatory and compulsory are often used interchangeably, following Navin and Largent (2017), I will use mandatory to refer to measures that do not involve the criminalization of refusal.

Implementing a vaccine mandate makes it clear that vaccination is not a personal choice, but rather something expected as a member of a population, similar to taxes (Giubilini, 2020). Mandates are generally aimed at those who are vaccine-hesitant rather than those who are staunchly opposed to vaccination (Pierik, 2018). By generating a social norm of vaccination, making vaccine refusal costly or inconvenient, and by providing assurance that others are also making their contribution, mandates can improve vaccine uptake rates. Several studies in different settings have shown mandates to be effective in increasing vaccination uptake (Robbins *et al.*, 1981; Orenstein and Hinman, 1999; D’Ancona *et al.*, 2019; Lévy-Bruhl *et al.*, 2019).

Mandatory vaccination exists in some form in most countries, most commonly for childhood vaccine preventable diseases (Gravagna *et al.*, 2020). For childhood vaccines, mandates have taken the form of requiring vaccination for the receipt of some welfare benefits (in Australia), attendance at state-run school or childcare centres (the USA and Italy). In adults, instances of mandatory vaccines have included requiring health care workers to receive certain vaccines in order to work with patients (Field, 2009).

The Nuffield Council on Bioethics’ 2007 report on ethical issues in public health suggests that when considering whether directive vaccine policies are acceptable, the following should be taken into account: the seriousness of the threat to the population, and the risks associated with the disease and vaccination itself (Nuffield Council on Bioethics, 2007). The report suggests that ‘quasi-mandatory’ policies are more likely to be appropriate for diseases that are highly contagious and serious, and diseases where eradication may be possible. More recently, Julian Savulescu has argued that mandatory vaccination may be permissible when four conditions are met: (i) that the disease is a grave public health threat, (ii) that there is a safe and effective vaccine, (iii) mandatory vaccination has a superior cost/benefit profile compared with other alternatives and (iv) the level of coercion is proportionate (Savulescu, 2020).

## Mandatory Vaccination in COVID-19

It seems relatively clear that COVID-19 poses a serious threat to the health of many countries’ populations. In the first 12 months of the pandemic, in the UK, over 100,000 people had died and had COVID-19 on their death certificate (Public Health England, 2021) and modelling suggests unmitigated epidemic would result in hundreds of thousands of deaths in the country (Ferguson *et al.*, 2020; Ragonnet *et al.*, 2020). Aside from this direct mortality impact, many survivors will suffer significant morbidity, and an unmitigated epidemic would likely overwhelm health care services, jeopardizing health more broadly (Ferguson *et al.*, 2020). At least in the UK and nations like it, COVID-19 does seem to present a grave public health threat.

Vaccine development has been faster than many initially predicted, and several vaccines were developed during 2020. Although some vaccines have been associated with rare side effects (discussed further below), the risks of the vaccines are low, and they have been authorized for use in many countries and by the World Health Organization (2021). In many settings voluntary COVID-19 vaccine uptake has been high. Within the first 6  months of vaccine availability, the proportion of people over the age of 65 who received at least one dose of vaccine was >90% in the UK (Office for National Statistics, 2021) and >85% in the USA (US Centers for Disease Control, 2021a) and several European countries (European Center for Disease Control, 2021). However, vaccine uptake among high-risk groups has been much slower in some locations, and this rate of uptake is important for minimizing the overall harms of the pandemic. There is heterogeneity in uptake within countries, with uptake being well below average in some areas, leaving some communities vulnerable to ongoing outbreaks which may threaten local health care systems. Indeed, in September 2021, 10 months after vaccines became available, some areas of the USA were reported to be rationing health care due to COVID-19 surges (Boone, 2021). In October 2021, in 26% of US counties, fewer than 70% of people aged 65 years and over had received at least one vaccine dose. In 10% of counties fewer than 30% of this age group had received at least one vaccine dose (US Centers for Disease Control, 2021b).

Slow vaccine uptake has seen several states in the USA implement incentives for vaccinations, such as cash or lotteries (Knutson, 2021). Strategies to increase vaccine uptake are clearly needed, and may be even more important for booster vaccinations. Given that the longer the COVID-19 pandemic continues, the greater the harms caused, taking an approach of ‘waiting and seeing’ whether vaccines are taken up sufficiently rapidly voluntarily may carry significant costs. The potential health gains of a selective mandate provide a strong argument for consideration in policy.

One important feature of COVID-19 is the heterogeneity in disease severity across population groups. While a variety of factors influence risk of severe COVID-19, the greatest risk comes with older age (Williamson *et al.*, 2020). The infection mortality ratio for SARS-CoV-2 (the causative pathogen of COVID-19) is lowest in young children, with median estimates between 0.01% and 0.001% for those aged 5–9 years (Brazeau *et al.*, 2021), but rises in a log-linear pattern among people aged 30 years and over. For those aged over 80 it is estimated to be 8.29% (O’Driscoll *et al.*, 2020), although even in this group the risk continues to increase dramatically with age, with one estimate putting the risk at around 5% for those aged 80–84 and 17% for those 90 years and older (Brazeau *et al.*, 2021). A modelling analysis (Ragonnet *et al.*, 2020) has suggested that if those at highest risk of disease could be effectively isolated, then even if infection were allowed to occur in younger people, the mortality costs of the pandemic would be drastically reduced. Of course, this does not take into account health impacts apart from mortality, but morbidity from COVID-19 is also higher among older people, with long-term harms correlated with disease severity, and long-covid also more likely among those who are older (Sudre *et al.*, 2021). This heterogeneity in risk of harm is relevant to discussions of mandatory vaccination. It suggests that vaccinating this high-risk subset of the population may cause disproportionate public health benefit and that the risk–benefit profile of vaccination will be different for different groups. As such, it may be the case that mandatory vaccination is only appropriate for high-risk age groups.

This paper explores the idea of selective mandatory vaccination in the setting of COVID-19. In future pandemics the characteristics of the disease in the population may be different, and it may be different groups that have higher risk of severe disease. For example, in the 1918 influenza pandemic it was younger adults (aged 25–40) who were at highest risk of severe disease (Liang *et al.*, 2021). Rather than disease severity, transmissibility may be the key factor that varies among groups. In previous influenza pandemics children have been significant vectors for transmission, and it has been argued that a COVID-19 vaccine mandate for children may be permissible if it were the case that children are particularly important vectors for transmission (Savulescu *et al.*, 2021). The groups targeted by selective mandate may therefore be different depending on characteristics of the disease in a population.

Although it is possible that new variants may, in the future, change the epidemiological features of COVID-19, at this point in time there is no evidence to suggest that new variants have substantially changed the demographic patterns of disease severity or transmissibility (Lewis, 2021). Therefore, the following argument proceeds with the assumptions that age is a particularly important risk factor for disease severity and that there is no demographic group that is particularly important for transmission. However, changes in these epidemiological features, should they occur, will alter the applicability of this argument.

## Optimizing Outcomes and Removing the Grave Public Health Threat

One of the reasons to consider mandatory vaccination is the potential health and well-being gains it might produce. Indeed, the Nuffield Council’s reference to the ‘seriousness of the threat to the population’ and Savulescu’s reference to ‘grave public health threat’ appeal to the magnitude of health and well-being that is at risk without vaccination.

Ultimately, vaccinating the entire population against COVID-19 would best minimize direct COVID-19 morbidity and mortality. This would protect those who cannot be vaccinated, and better protect those at risk of severe disease, as no vaccine is 100% effective. It would also protect the younger population. Although the mortality risk is relatively low for this group, it is not zero. COVID-19 also poses health risks apart from death, including the harm of the acute illness, long-Covid, and possibly unknown long-term effects. All else equal, it is better for everyone to avoid contracting COVID-19.

However, time is crucially important in the COVID-19 pandemic, and vaccines cannot be produced and distributed to everyone immediately. Strategies need to take into account what will lead to the best outcomes given the immediate constraints on vaccine distribution. Modelling studies have suggested that in this condition of scarcity, prioritizing the elderly for vaccination is most likely to minimize COVID-19 mortality (Hogan *et al.*, 2020; Bubar *et al.*, 2021; Moore *et al.*, 2021). This is unsurprising given that those aged over 65 account for the majority of COVID-19 deaths: 92.5% in England (Office for National Statistics, 2020). Choices around vaccination programs may be influenced by several different values, including saving lives, saving life years, saving quality-adjusted life years, protecting the health care system or restoring the normal functioning of society (Giubilini *et al.*, 2021). It is therefore not clear that the goal of a vaccination program ought to be minimizing the number of deaths. However, it is likely that protecting those most at risk of severe disease would minimize the mortality burden in terms of both number of deaths and years of life lost. As this group is also the most likely to require health care resources, this would also be likely to most effectively prevent the health care system from being overwhelmed. This also suggests that the ‘grave public health threat’ would be substantially reduced, and possibly eliminated, if this group were protected with an efficacious vaccine. From a population health perspective ensuring rapid uptake of the vaccine in this group, and others at high risk, should be an immediate priority.

## Justifying Mandatory Vaccination: The Harm Principle

Even if mandatory vaccination were only considered for those most at risk and who would clearly receive substantial individual benefit from COVID-19 vaccination, a justification still needs to be provided as to why their autonomy can be overridden. People often choose options that may not seem to be best from them, and this freedom to choose is highly valued in many societies. Here John Stuart Mill’s suggestion that the state can only restrict a person’s liberty to prevent them from causing harm to another (Mill, 2011) has been influential.

Indeed, existing discussion of mandatory vaccination commonly appeals to this ‘harm principle’. Vaccination not only protects the vaccinated individual but also prevents them directly harming others by passing on infection, and indirectly harming others by unnecessarily requiring health care resources. Giubilini and Savulescu (2019) liken the duty to vaccinate oneself to the duty to wear a seatbelt. In both cases the action prevents direct harm (to other vehicle occupants or to people to whom an infection may be transmitted), as well as indirect harm to other health care service users, and this risk of harm overrides the individual’s autonomy to choose the riskier option for themselves. Similarly, Flanigan (2014) describes mandatory vaccination as akin to forcibly preventing someone from firing a gun into a crowd. She suggests that in the same way that we think it is permissible to override a person’s autonomy to prevent them from firing a gun and risking harm to others, we should consider it permissible to override autonomy to ensure people are vaccinated to prevent them harming others.

While the harm principle provides an effective basis for when it may be ethical to override a person’s autonomy, judgement is required to determine circumstances where this is proportionate. Many things we do pose a risk of harming others, for example driving a car poses a risk to other motorists and pedestrians. So, it must be shown that the risk of harm is sufficiently high, and the cost of removing it proportionately low. Building on Flanigan’s analogy, Giubilini points out that when a population is close to herd immunity for a particular disease, an individual failing to vaccinate only adds a minor risk of harm to others. As he says, in such cases mandatory vaccination may be more like preventing someone from firing a gun when everyone is wearing bulletproof vests (Giubilini, 2020). In this instance, the argument from the harm principle is not as strong. In the case of COVID-19, if those at high risk were already vaccinated, then vaccinating people at low risk would be like preventing them firing a gun whose bullets only harm a subset of the population, and that subset are wearing bulletproof vests. If those at high risk from COVID-19 were all protected through vaccination, the argument that others remaining unvaccinated poses significant harm is weakened.

On the other hand, the harm principle does support mandatory vaccination of those who are at high risk of severe COVID-19. As mentioned, the indirect harms caused by the COVID-19 epidemic are substantial, both through disruption to health services and the limitations on public movement. Analyses have suggested substantial harms from disrupted cancer screening services (Maringe *et al.*, 2020) and from school disruption (Christakis *et al.*, 2020), as well as mental illness exacerbated by lockdown measures (Pierce *et al.*, 2020). In the UK, population movement restrictions have been justified by appealing to the need to save lives and protect the National Health Service (NHS). The same justification applies to vaccination, especially for those at risk of severe disease that puts greater strain on the NHS. For those at risk of severe disease, the harm principle suggests that it is permissible to override their autonomy on the decision of whether to receive the COVID-19 vaccine, as their risk of infection carries a risk of using health care services unnecessarily, and prolonging socio-economic disruption with its attendant health costs.

In instances where one group is a particularly important vector for transmission then this increased risk of harming others (and thereby also indirectly contributing to health care systems becoming overwhelmed) would be a relevant feature for considering the permissibility of a vaccine mandate. The magnitude of the risk posed by this group may be sufficient to implement a mandate, where it may not be warranted in others who pose a lower risk of transmission. Thus far, no particular demographic group has been identified to be particularly key transmitters of COVID-19, but it is possible that such a group would be identified in the future. Mandating vaccination for this group may be justifiable given certain conditions, but further ethical analysis would be required.

## Fair Contribution to a Public Good

Another consideration of any public health policy option is the implications for fairness. Giubilini (2020) suggests that mandatory vaccination to achieve herd immunity in childhood diseases may be justified by appeals to fairness, rather than simply appealing to harms. By likening vaccination to taxation, he suggests that population immunity is a public good and the measures taken to reach this public good should be fairly distributed. In the case of COVID-19, given immediate vaccine scarcity the most important public good from vaccination is rapid epidemic control and removal of the grave public health threat, rather than herd immunity.

Here again the heterogeneity in risk for COVID-19 has implications, as the risk–benefit profile of vaccination compared to the risk of the disease is different for different groups. It is less costly for those who are most at risk of COVID-19 to be vaccinated, as the risk–benefit profile of individual vaccination is more clearly in favour of vaccination. For those at lower risk of severe disease vaccination asks them to accept a less favourable risk–benefit profile.

This difference in risk–benefit profiles is important, as it affects the level of burden that an individual is being asked to take on as a contribution to the public good of removing the public health threat of COVID-19. Although for people at high risk of severe COVID-19 vaccination is very clearly in their interests, the reason to implement a mandate is not to force people to protect themselves for their own sake, but rather is to reduce the risk of their illness contributing to overwhelming the health care system and/or requiring prolonged population-movement restrictions. So even though they are the primary beneficiary of the vaccine, rather than being coerced to vaccinate for their own benefit, they are being coerced to vaccinate for the public good. Kraaijeveld (2020) has developed a framework for differentiating types of vaccination based on who makes the decision to vaccinate and who is the primary beneficiary of the vaccine. On this framework, a COVID-19 vaccine mandate for those at highest risk might initially seem to be an instance of paternalistic vaccination, as the decision is made by someone other than the vaccinee and the vaccinee is the primary beneficiary of the vaccine. However, even though the vaccinee does receive a large benefit from the vaccine, the reason to coerce them to vaccinate is actually to protect the interests of others by removing the public health threat and thereby preventing the societal harms of an uncontrolled epidemic. So, this is either an instance of what Kraaijeveld calls indirect vaccination (where someone other than the vaccinee makes the decision to vaccinate for the purpose of bringing about benefit to others), or the scenario may fall outside the scope of Kraaijeveld’s framework. It is not paternalism, but rather contribution to the common good and prevention of indirect harms to others, that is the motivation for the mandate.

For most adults, a COVID-19 vaccine is very clearly in their interests. Whatever small risks the vaccine may have, these are vastly outweighed by the reduction of risk from COVID-19 that the vaccine provides. However, at this relatively early stage of the vaccine’s use, for some younger age groups, we cannot be so confident that the risk–benefit profile of the COVID-19 vaccine leads to vaccination being clearly in the individual’s clinical interest. This is highlighted by several countries introducing limits on the type of vaccine used in younger age groups (Gallagher, 2021; Olsen, 2021). Vaccines often can cause short-lived adverse effects such as fatigue, malaise and pain, but also carry a small risk of more severe side effects, including anaphylaxis and other complications. The phenomenon of vaccine-induced immune thrombotic thrombocytopenia, and more recently evidence suggesting a link between myocarditis in younger males and some mRNA vaccines, has highlighted how the risk–benefit profile of a vaccine can vary depending on demographic features (Greinacher *et al.*, 2021; Vogel, 2021; Winton Centre for Risk and Evidence Communication, 2021). It has also highlighted how vaccines (and other medical interventions) can have unexpected risks that may not be detected in clinical trials. This highlights the importance of another feature that distinguishes COVID-19 from other vaccine preventable diseases—its novelty, and the uncertainty in disease and vaccine risk profile that entails. Studies assessing childhood vaccines have not found evidence of long-term harms (Pittman *et al.*, 2004), but there is a risk that new vaccines may have unexpected harms. For example, a vaccine developed for the 2009 influenza pandemic was associated with a small risk of developing narcolepsy in children (Miller *et al.*, 2013). For most vaccines there is a long history of use and substantial data monitoring for long-term harms, which allows us to make confident statements on the nature of their risks, such as this from the UK’s NHS: ‘vaccines get safety tested for years before being introduced—they're also monitored for any side effects’ (National Institutes of Health, 2020). This statement cannot be made for any COVID-19 vaccine. These possible risks of long-term harm are also more important for younger people than older people, as few safety trials have involved young people (children and adolescents), and because the young can expect to live longer to experience them if they do occur, and for their effects to affect a greater portion of their life. This inability to provide confidence in the risk–benefit profile, particularly for those at low risk from COVID-19, makes vaccination a comparatively greater cost to people at lower risk, especially younger people, than it does to older people at higher risk of disease.

If it were the case that the most effective way to remove the public health threat of a disease was to target those who are most likely to transmit the disease, rather than those most at risk of severe disease, then appealing to the idea of fair contribution to a public good will be more complicated. Consideration of fairness may instead be a reason against pursuing that approach if it asks those who can contribute most to removing the public health threat to take on an overly large burden. However, given modelling has consistently recommended vaccinating those who are most vulnerable to severe COVID-19 first, in this situation efficiency and fairness coincide, and consideration of fairness adds to the argument to mandate a vaccine for those at highest risk of severe COVID-19.

In some instances it may be appropriate to ask people to accept an unfavourable risk–benefit profile for the purposes of protecting others. Previously it has been argued that children should be vaccinated against influenza for the primary purpose of protecting the elderly (although in this case the risk–benefit profile clearly favours vaccination for children as well) (Bambery *et al.*, 2018). However, much of the strength of the argument rests on the vaccine not providing substantial protection directly to the elderly, and children being an especially important vector for transmission. It has also been argued that should these conditions hold for COVID-19, then it would likely be ethically acceptable to vaccinate children against COVID-19 in order to protect older people, provided that the risk of vaccination is sufficiently small (Giubilini *et al.*, 2020; Savulescu *et al.*, 2021). However, we now have more information on COVID-19 and vaccines. The evidence suggests that COVID-19 vaccines have comparable efficacy across age groups, including older adults (Anderson *et al.*, 2020; Folegatti *et al.*, 2020; Pfizer, 2020). So current evidence suggests that the elderly will have substantial protection from being vaccinated themselves. Although the role of children in transmission remains an area of uncertainty, the evidence that is available does not suggest that children are a particularly important vector for COVID-19 transmission (Dattner *et al.*, 2021; Munro and Faust, 2020). Combined with the uncertain risk–benefit profile to low-risk individuals and the uncertain effect of COVID-19 vaccination on transmission risk, it is less clear that the small level of additional protection for the elderly arising from vaccinating low-risk people justifies the infringement of autonomy and small risk imposition required by mandatory vaccination.

It may also be argued that, although the clinical risk–benefit profile of vaccination may not be clearly favourable for younger people, the indirect benefits from the resolution of the COVID-19 crisis will tip the balance to be clearly in favour of vaccination for younger people as well. Indeed, it might be suggested that younger people stand to gain more from removing the public health threat of COVID-19 than do older people, as they may suffer more due to the pandemic’s socio-economic effects, which will likely persist for many years into the future. Concern for fair contribution to a public good might then suggest that younger people ought to take on more of the burden of achieving epidemic control, even though their clinical risk–benefit profile is lower, as they stand to gain more from this good. However, making this comparison of who stands to gain more from rapid resolution of the crisis is difficult. It could equally be argued that older people have a greater interest in achieving rapid resolution of the pandemic as they have fewer years remaining, so it matters more to them how much of the next few years are spent with restrictions. These sorts of considerations also raise difficult and controversial philosophical questions on the nature of personal identity and how this persists through time. Considering the clinical risk–benefit profile of vaccination provides a clear idea of who is being asked to take on what degree of risk.

Furthermore, if, as the modelling suggests, the fastest path to resolving the crisis is through vaccinating those at highest risk of severe disease, then we ought to take this path, which involves minimizing the number of people who are asked to accept a less clearly favourable clinical vaccination risk–benefit profile. In a setting of global vaccine scarcity, removing the grave public health threat everywhere should be the immediate priority, rather than reaching herd immunity in a few settings. Globally, at least initially, COVID-19 vaccines are going to be a scarce resource. So, if one country was in a position to procure a greater portion of the global vaccine stock for their population, a strategy that relies on larger quantities of vaccine will delay the access of another country to the vaccine, costing lives there. This suggests that achieving epidemic control through rapid vaccination of those at highest risk, rather than waiting for population immunity to develop through voluntary vaccination is also a matter of global justice.

## Proportionality and Considerations for Policy

I have thus far argued that a selective mandate could be an ethical policy choice. However, the form of the mandate would need to maintain respect for, and limit the burden imposed on, the individual, and prevent population-level harms from the mandate itself. The costs imposed by the mandate should act as sufficient disincentive to non-compliance, so that vaccination for these groups is seen as something expected, rather than an option that is the decision of the individual alone. The form of the mandate, and the level of coercion involved, needs to be proportionate.

As mentioned, mandatory vaccination does not imply criminalization of vaccine refusal (Navin and Largent, 2017) but may involve other measures like fines, community service or movement restrictions. For example, people at high-risk of COVID-19 who refuse vaccination could be required to remain in isolation (or otherwise have their freedom of movement restricted) until epidemic control is achieved, to reduce their risk in another way. If there were concerns that even this level of cost would unduly burden some people (for example residents of nursing homes who are often already deprived of social contact), then exemptions could be included. Alternatively, the cost of non-compliance could be reduced even further, so that the mandate only created a minor inconvenience (e.g. completing and submitting forms for exemptions). The purpose of the mandate is to make non-compliance costly, so that more of those who are hesitant end up being vaccinated, rather than enforcing compliance among the entire population. However, reducing the cost, or increasing the number of people who are exempt, would reduce the effectiveness of the mandate. A choice would need to be made on how to trade off effectiveness with a level of cost that seems proportionate and unlikely to cause excessive harms.

Alternatives should also be considered, including a payment model, which has been suggested by Savulescu (2020). This may be a better option in some settings. However, like the ‘wait and see’ approach, this too carries risk that the payment will not be a sufficient incentive to achieve rapid uptake. Use of local data and community involvement may help to determine the role of a selective mandate in a vaccination plan, and the best option for implementation, to ensure fairness, effectiveness and respect for the population. It would also be important to establish a compensation mechanism for those who suffered adverse events as a result of mandatory vaccination (Savulescu *et al.*, 2021).

## Objections

I have thus far argued that mandatory COVID-19 vaccination for those at high risk of severe disease may result in substantial welfare gains and is justified by the harm principle and the value of fairness. However, it could be argued that mandating vaccines risks strengthening anti-vaccine sentiments, and that selective vaccination is discriminatory. I will respond to these objections now.

### Does Mandatory Vaccination Risk Strengthening the Anti-Vaccine Movement?

It has been suggested that mandatory vaccination carries risks of undermining public trust in vaccines (Omer *et al.*, 2019). Given the high profile of the COVID-19 vaccine, any adverse events in vaccine recipients are likely to be highly publicized. As vaccines do carry small risks, there will be (and have already been) cases of serious adverse events. Furthermore, it is also likely that there will be instances of people suffering an acute health emergency shortly after receiving the vaccine, and even if this is not due to the vaccine, it will be difficult to separate the events in the eyes of a sceptical public.

On the background of this scrutiny, mandated vaccination may have the potential to further undermine public confidence in vaccines. However, the risk seems lower if vaccination were only mandated in those at highest risk. The most outrage-inducing scenario would be a young person, at low risk of COVID-19, who suffers, or appears to suffer, an adverse event due to a mandated vaccine. If an older person at higher risk of COVID-19 were to suffer an unexpected adverse event it would still be tragic, but a mandate would be more easily justified due to their higher ex-ante risk of COVID-19. If an elderly person were to suffer an unrelated health event soon after their vaccination, it would also be less likely to be mistakenly attributed to the vaccine, as such events are not as unexpected in older age.

The risk of undermining public confidence in vaccines needs to be balanced against the harm of a longer COVID-19 pandemic. Mandatory vaccination of only those at highest risk of severe disease seems to strike the right balance between these two risks: avoiding the risk of the most outrage-inducing scenario, while also providing the majority of the public health benefit of rapid vaccine uptake.

### Is a Selective Mandate Discriminatory?

It may be argued that the selective mandate I have proposed constitutes unjust discrimination on the basis of age. However, as Savulescu and Cameron (2020) have argued, risk of severe disease, which in the case of COVID-19 correlates with age, is a morally relevant factor in health policy. To discriminate on the basis of a morally irrelevant factor would be unjust. But discriminating on the basis of a factor that has significant implications for the public health utility of the measure, as well as the individual’s own capacity to benefit, is not unjust. To borrow an example from Savulescu and Cameron, offering breast cancer screening to women and not men doesn’t constitute unjust discrimination on the basis of sex, as women are much more likely to benefit from breast cancer screening.

More recently, Cameron *et al.* (2021) have argued that selective liberty restrictions may be appropriate when such restrictions would bring benefits for both the population and the individual, are designed to protect those most at risk of disease, and are no more than is necessary. They also specify that differences that arise due to social disadvantage itself ought not to be the basis for discriminatory health measures. This is designed to promote respect for, and protect the interests of, the individual as well as promote the interests of the population.

In the case of COVID-19, as well as age, race has also been correlated with disease severity (Sze *et al.*, 2020). For example, in the USA, Black and Hispanic ethnicity has been associated with substantially greater risk of death from COVID-19 (Ford *et al.*, 2020). However, there is evidence that the discrepancies are largely due to social disadvantage, rather than race per se, as in some analyses, when discrepancies in access to health care and social disadvantage are taken into account there is no significant difference in mortality outcomes due to race (Iacobucci, 2020; Lopez *et al.*, 2021). In this case a mandate on the basis of race would be roughly approximating a mandate on the basis of social disadvantage, which generates new ethical problems. Even if race were an independent contributor to risk, there are other reasons why a selective mandate on the basis of race would be ethically problematic. On a background of historic and ongoing racism in medicine (Nuriddin *et al.*, 2020), mandating vaccination on the basis of race would raise further important ethical problems than have been considered here, and may lead to important negative consequences such as heightening racism in medicine, reducing trust in medical institutions, increasing racial tensions and augmenting inequalities. Introducing mandates on the basis of race would also be practically difficult, given the variation in racial identities that exist.

Although ageism exists in society, a mandate based on age would be less fraught with social tensions. There are many precedent examples of selective public health measures based on age; most vaccine schedules are based on age, as are many screening interventions such as breast or bowel cancer screening. Furthermore, ageing is a universal experience, so although birth cohorts can be distinguished, all of us (unless we die prematurely) will experience ageing, meaning that measures based on age involve less inequality when considering the course of a life. A mandate using age would also be practically easier, given the clear availability of age data in most settings.

## Conclusion

Rapid uptake of a safe and effective COVID-19 vaccine in those at highest risk of severe disease will be important to mitigating the impact of the pandemic. Although vaccination is likely to be in the interests of most people, making a vaccine mandatory involves impositions on autonomy which need to be justified. The infringement of autonomy of those at high risk of severe COVID-19 is justified on grounds of the harm principle and also promotes fairness. However, if those at high risk were protected, there is little grounds to justify mandating vaccination for those at lower risk of severe COVID-19, particularly in the early stages of vaccine use. Although a selective mandate may be an ethical choice, the form of the mandate would need to be proportionate and avoid being overly costly for individuals or worsening inequality. Imposition or prolongation of movement restrictions may be an appropriate measure. Whether a selective mandate should form part of a COVID-19 vaccination plan, and if so, the form that it takes, is a larger question that may have different answers depending on context. A mandate alone is unlikely to be the best approach, with attention needing to be paid to communication strategies, and identifying and eliminating barriers to vaccine uptake. However, based on current evidence of disease risk and transmission, a selective mandate for the COVID-19 vaccine on the basis of age is likely to be ethically justifiable and, in the face of real and important harms from vaccine hesitancy, policymakers should give careful thought to the role it may play in vaccination strategies.
